# Preparing Community Health Workers to Empower Latino(a)s With Diabetes: A Real-World Implementation Study

**DOI:** 10.1177/26350106231220012

**Published:** 2024-01-20

**Authors:** Laura Porterfield, Zuleica Santiago Delgado, Premal G. Patel, Michael L. Goodman, Kendall M. Campbell, Elizabeth M. Vaughan

**Affiliations:** Department of Family Medicine, University of Texas Medical Branch (UTMB), Galveston, Texas; Sealy Institute for Vaccine Sciences, UTMB, Galveston, Texas; Department of Family Medicine, University of Texas Medical Branch (UTMB), Galveston, Texas; Department of Internal Medicine, UTMB, Galveston, Texas; Department of Internal Medicine, UTMB, Galveston, Texas; Department of Family Medicine, University of Texas Medical Branch (UTMB), Galveston, Texas; Department of Internal Medicine, UTMB, Galveston, Texas; Department of Medicine, Baylor College of Medicine, Houston, Texas

## Abstract

**Purpose:**

The purpose of the study was to evaluate the delivery of diabetes self-management education (DSME) to Latino(a) adults by community health workers (CHWs).

**Methods:**

Investigators developed an evidence-based, bilingual (Spanish/English) diabetes education curriculum and trained 10 CHWs on its content. CHWs then implemented the curriculum in 6-month diabetes group visit programs for low-income Latino(a)s with type 2 diabetes in nonacademic 501(c)3 community clinics. Investigators evaluated efficacy of the training through successful implementation, measured by participant group visit acceptance and attendance.

**Results:**

Participants (n = 70) reported high levels of program satisfaction (3.8/4.0), improvement in quality of life (9.7/10), meeting of individual needs (3.8/4.0), and acceptability (9.7/10.0). Content analyses revealed that 87.1% of participants would not change the program or wanted to extend it. Participant attendance was 81.6%.

**Conclusions:**

Investigators demonstrated the ability to develop a training that nonmedical personnel (CHWs) successfully implemented in a real-world study. This study provides a curricular framework for CHW-led education that may serve as a template to extend to other diseases and populations.

A total of 70 million Americans rely on uncompensated or publicly funded medical care,^1^ creating an urgent need for resource-saving and innovative approaches for achieving quality care. One such innovative approach to improving care and achieving health equity is integrating community health workers (CHWs) into community health centers (CHCs) and other health care teams.^2^ The American Public Health Association defines CHWs as individuals with close ties and rapport with the community they serve.^3^ Although CHWs often lack formal medical education, they engage in a variety of health-promoting functions, including participant education.^1,4,5^ Studies have shown that CHWs are efficacious and cost-effective in improving care quality and reducing staff burden in resource-limited settings.^3,5,6^ For example, a recent large (n = 5649) quasi-experimental study of Mexican Americans with type 2 diabetes revealed that CHW-delivered programs were associated with improvement in diabetes outcomes.^7^ CHW-associated improvements are often credited to CHWs’ shared ethnicity, culture, language, and life experiences with the participants they serve, which likely contribute to enhanced primary care.^8,9^ CHWs are critical to the implementation of high-quality care in their communities and are a vital part of the health equity landscape.^10^

Despite the promise CHWs show for improving outcomes and equity for people and communities facing barriers, there is not a national consensus on their certification and training. This has resulted in lack of standardization, threatening the ability to fully evaluate CHW efficacy and potentially posing a risk to patient safety.^11-13^ As a result, structured education, training, and support of CHWs, tailored to their intended role and context, have been identified as priorities in CHW deployment.^9,11^ Strengthening the training and curricula available to CHWs can empower them to become collaborative leaders in the promotion of health while decreasing the cost and labor burden of medical services.

In the previously described TIME (Telehealth-supported, Integrated CHWs, Medication access, group visit Education) studies, investigators developed a curriculum for nonmedical personnel (CHWs) to teach diabetes group visits for low-income Latino(a) participants.^14-17^ TIME CHWs received competency-based, Category 1 medical training (ie, Health Insurance Portability and Accountability Act, protected health information, and teaching strategies).^18^ Investigators also provided CHWs with Category 2 and Category 3 disease-specific training on diabetes that included the same curricula that CHWs would teach to participants. Investigators previously reported the clinical value of this intervention, including improved A1C, blood pressure, and preventive care measures that were sustained at least 24 months.^14-17^ However, investigators have not reported on TIME’s curricula or a descriptive analysis of its acceptability among participants. Therefore, the aim of this study is to evaluate the delivery of diabetes self-management education (DSME) to Latino(a) adults by CHWs. Investigators anticipate that future investigators will be able to utilize these step-wise curricula for other diseases and conditions, providing more real-world implementation of CHW initiatives with standardized training. This is a critical piece needed for a national consensus on CHW training and certification.

## Methods

### Research Design

The TIME project was conducted as a randomized clinical trial at nonprofit clinics serving uninsured, low-income (earning ≤250% federal poverty level) individuals in the greater Houston, Texas, area. Detailed methodology and clinical outcomes of this trial are reported elsewhere.^14-17^

### CHWs, Participants, and Setting

Investigators reported on 4 TIME participant cohorts recruited from 2018 to 2021 at CHCs in the Houston area serving low-income individuals without insurance. Group visits met monthly for 6 months and were conducted in Spanish. CHWs taught 30-minute large group (n = 20-25) sessions. Participants then participated in three 30-minute small group breakout sessions (n = 6-9/session), totaling 90 minutes. The breakout sessions included 1 clinician-led medical management group and 2 CHW-led social and behavioral sessions.^14-16^

Investigators recruited participants from CHCs using a clinic database to screen for participants with type 2 diabetes (ICD-10 E11.X). Individuals with type 2 diabetes were contacted to participate if they met the following inclusion criteria: self-identified Latino(a)/Hispanic adults (≥18 years). Exclusion criteria included pregnancy, type 1 diabetes, inability to receive care in a group setting due to severe mental illness or high level of disease complexity, and any condition that could alter A1C levels, such as a recent blood transfusion. CHWs were self-identified Latino(a)s, fluent in Spanish, and certified in the state of Texas (certification: 160-hour training program or 1000 community hours; recertification: 20 hours of continuing education biennially). The Baylor College of Medicine Institutional Review Board approved the study. All participants provided written informed consent.^14-16^

### Theoretical Model and Framework for the Study

The framework underpinning the TIME project is the chronic care model (CCM). CCM involves a redesign in care for patients with chronic diseases, shifting from the prevalent US model with its focus on acute care and passive patient involvement to a primary-care-based model in which teams proactively anticipate the needs of activated and informed patients.^19,20^ The 6 components of the CCM include clinical information systems, delivery system design, decision support, self-management support, organization of health care, and community resources.^20^ The TIME project drew on these pillars by restructuring the approach to diabetes management through group visits and provider and CHW education on evidence-based practices (delivery system design, organization of health care, clinical information systems, decision support) and by bolstering the team and strengthening community connections through the incorporation of CHWs (community resources, self-management support, delivery system design).^19,20^ This structure not only transformed clinical sites into care teams empowered to proactively address chronic disease needs but also equipped the teams to activate and inform patients for engagement in their care and health.

### Curricular Context and CHW Training

The research team developed the participants’ curriculum. The curriculum was structured on the core competency and workforce framework derived from the CHW Core Consensus Project (CCCP).^11,18^ In the CCCP, a national expert panel identified 3 categories of CHWs based on training, practice setting, and scope, with higher categories associated with increased specialization.^11^ The CCCP also identified 6 domains of CHW core competencies, offering a practice-oriented and quantifiable system for CHW education.^11^

This team included the study principal investigator (PI; physician) and co-investigators with expertise in behavioral health, implementation research, and endocrinology. To create the curriculum, investigators used evidence-based literature, the FICA evaluation (Faith, Influence, Community, Address), and standards of care from the American Diabetes Association, Joint National Commission (hypertension management), Adult Treatment Panel (cholesterol management), and US Preventive Task Force.^21-30^ The curriculum consisted of 6 PowerPoint modules designed for teaching large groups of low-income participants with type 2 diabetes and questions for small group breakout sessions. Breakout session questions addressed social and behavioral barriers to the monthly large group topic. Each group included 3 questions per month to define the problem, discuss the solution, and set goals.

Table 1 provides an overview for the program, including the large and small group topics. Although each module is freestanding, Modules 1 and 2 are presented first to provide fundamental disease concepts. The order of the latter 4 modules may vary depending on participants’ needs and interests. English and Spanish versions of the PowerPoint modules and small group breakout session curriculum are available on the project website (mipromotordesalud.org).^31^

**Table 1.** Curriculum Overview for a Community Health Worker-Led diabetes program

Month	Large Group Topics and Objectives	Social Barriers	Behavioral Barriers	Corresponding Chronic Care Model Component(s)^19^
1	Diabetes overview - Define diabetes and A1C goals - Discuss prevention of complications	- Taking ownership - Glucometer teaching	Fear and faith	- Clinical information systems - Self-management support
2	Medication adherence - Identify adherence barriers and approaches to overcome them - Discuss medication rationale - Define normal blood sugar	Taking your medications	Life’s influences	- Delivery system design - Clinical information systems
3	Nutrition - Review importance of nutrition - Explore barriers to good nutrition - Discuss how to simplify nutrition - Set nutrition-related goals	MyPlate.gov activities	What matters: importance	- Decision support - Self-management support
4	Sex/intimacy & depression - Review diabetes-related complications relating to sexual intimacy and depression - Discuss importance of glucose control	Diabetes sequelae	Being part of a community	- Self-management support - Decision support
5	Preventive care - Review rationale for preventive care - Discuss how to prevent diabetes complications - Review age-appropriate preventive care	Age-appropriate guidelines	Community for coping	- Organization of health care - Self-management support
6	Exercise - Review importance of exercise - Explore barriers to exercise - Discuss how to simplify exercise - Set exercise-related goals	Hands-on exercise examples	Addressing your health	- Community - Organization of health care

The research team provided several layers of evidence-based training to prepare CHWs to lead the diabetes group visits.^26,28-30^ CHWs first received Category 1 five-hour entry-level training on the basics of teaching and working in clinics.^32^ CHWs then received disease-specific Categories 2 and 3 training using the same curriculum as was subsequently used for patient participants. CHWs also received ongoing telehealth-based training and support from a physician for 1-h/wk for the duration of the 6-month program. Ongoing training included effective teaching strategies, behavioral modification, and goal setting.^21-27^

### Evaluation Strategies

Investigators evaluated the efficacy of the CHW training by participant attendance and a descriptive analysis of participant satisfaction using survey feedback. Using a previously published survey,^14^ staff administered this 12-question survey to all participants at the end of the program (month 6) to evaluate program satisfaction (3 questions on 4-point Likert scale, where 4 is most satisfied), educator (CHW) satisfaction (4 questions on 10-point Likert scale, where 10 is most satisfied), and open-ended questions regarding what participants liked about the program, areas for improvement, and general feedback. Descriptive statistics were generated for this study. Investigators calculated mean values and corresponding standard deviations for numerical measures and conducted content analysis for the open-ended question responses. Investigators identified the top 3 themes in the responses of each open-ended question. Analyses were performed by a member of the research team with assistance from a non-team member. Any discrepancies were addressed with the study PI.

Investigators also evaluated CHW training by their training satisfaction outcomes. To evaluate training satisfaction, CHWs completed the 14-question Texas Department of State Health Services survey (12 questions on 6-point Likert scale, where 6 is excellent; 3 open-ended questions regarding usefulness of program, future topics, and additional comments).^33^

## Results

Table 2 shows baseline demographic characteristics of participants (n = 70). The majority were female (70%) with a mean age of 55.3 (±8.0) years. Participants averaged 12.9 (±8.4) years since diabetes diagnosis. At baseline, average A1C levels were 8.5% (±1.79), BMI was 32.7 (±5.62) kg/m^2^, systolic blood pressure was 133 (±17.7) mmHg, and diastolic blood pressure was 77.0 (±9.7) mmHg.

**Table 2.** Baseline Characteristics of Participants Across Cohorts in a Community Health Worker-Led Diabetes Program

Characteristic	Cohort 1 (n = 18) (% or ±SD)	Cohort 2 (n = 19) (% or ±SD)	Cohort 3 (n = 17) (% or ±SD)	Cohort 4 (n = 16) (% or ±SD)	Total (N = 70) (% or ±SD)
Sex (n)					
Male	1 (5.6)	6 (37.5)	7 (41.2)	7 (43.8)	21 (30)
Female	17 (94.5)	13 (68.4)	10 (58.8)	9 (56.3)	49 (70)
Age (y)	59.6 (±7.4)	52.8 (±5.1)	51 (±8.1)	57 (±9.0)	55.3 (±8.0)
Time since diabetes diagnosis (y)	18.8 (±9.8)	11.7 (±4.6)	8.6 (±8.5)	12.0 (±8.5)	12.9 (±8.4)
Employment					
Domestic	8 (44.4)	7 (36.8)	7 (41.1)	5 (31.3)	27 (38.6)
Maintenance	1 (5.6)	6 (31.6)	3 (17.7)	0 (0.0)	10 (14.3)
Food service	1 (5.6)	4 (21.0)	3 (17.7)	0 (0.0)	8 (11.4)
Unemployed	6 (33.3)	0 (0.0)	4 (23.5)	2 (12.5)	12 (17.1)
Other	3 (16.7)	2 (10.5)	0 (0.0)	3 (18.8)	8 (11.4)
A1C (%)	8.46 (±1.7)	9.55 (±2.2)	7.95 (±1.1)	7.59 (±1.2)	8.48 (±1.8)
BMI (kg/m^2^)	33.3 (±6.8)	31.7 (±5.4)	33.7 (±3.7)	32.0 (±3.7)	32.7 (±5.6)
Systolic blood pressure (mmHg)	135.8 (±10.4)	127.6 (±20.0)	134.3 (±13.4)	135.4 (±24.6)	133 (±17.7)
Diastolic blood pressure (mmHg)	75.5 (±7.3)	76.9 (±8.1)	77.1 (±13.0)	79.3 (±10.8)	77.0 (±9.7)

### Participant Results

Table 3 summarizes the quantitative results of participant feedback. There were 78 participants; 70 completed 6-month surveys and were included in the final analysis. Participants rated high levels of satisfaction: education/service received (3.8/4.0), individual needs met (3.8/4.0), and would come again for this education (3.8/4.0). Participants also reported that they would recommend the program (9.9/10.0) and that their quality of life improved (9.7/10.0). Participants were highly satisfied with the CHW-provided education (9.7/10.0) and found high value in their involvement in their health care (9.8/10.0).

**Table 3.** Curriculum Evaluation: Quantitative Analysis of a Community Health Worker-Led Diabetes Program

Evaluation Component	Cohort 1 (n = 18) (SD)	Cohort 2 (n = 19) (SD)	Cohort 3 (n = 17) (SD)	Cohort 4 (n = 16) (SD)	Total (N = 70) (SD)
To what extent did the diabetes classes meet your needs?^a^	3.6 (±0.6)	3.9 (±0.3)	3.7 (±0.5)	3.8 (±0.4)	3.8 (±0.5)
How satisfied are you with the service you received from the diabetes classes?^a^	3.6 (±0.8)	3.7 (±0.8)	3.9 (±0.2)	3.9 (±0.2)	3.8 (±0.6)
In the future, would you come back to these classes?^a^	3.7 (±0.6)	3.8 (±0.5)	4.0 (±0.0)	3.9 (±0.2)	3.8 (±0.4)
Calls or texts from the community health workers were helpful.^b^	8.9 (±2.1)	10.0 (±0.0)	9.9 (±0.2)	9.9 (±0.3)	9.7 (±1.2)
Having community health workers as part of your health care team was helpful.^b^	9.2 (±1.9)	10.0 (±0.0)	9.9 (±0.2)	9.9 (±0.2)	9.8 (±1.0)
I would recommend these classes to a friend or family member.	9.7 (±0.9)	10.0 (±0.0)	9.9 (±0.2)	9.9 (±0.2)	9.9 (±0.5)
My quality of life (comfort or happiness) is better because of these classes.^b^	9.1 (±1.1)	10.0 (±0.0)	9.9 (±0.2)	9.9 (±0.3)	9.7 (±0.7)

a Four-point Likert scale with higher score indicating more agreement.

b Ten-point Likert Scale with higher score indicating more agreement.

Table 4 summarizes the qualitative feedback from participants. Content analysis was performed to identify the top 2 or 3 themes in the responses to each open-ended question. For the first question (What did you like about the diabetes classes?), the top 3 themes were (1) the educational component (48.6%, n = 34), (2) characteristics and factors of the team delivering the classes (40.0%, n = 28), and (3) everything about the classes (52.9%, n = 37). For the second question (What would you want to change about the diabetes classes?), the top 3 themes were (1) no changes desired (77.1%, n = 54), (2) requests for more/longer intervention (10.0%, n = 7), and (3) adjustment of the session schedule (2.9%, n = 2). The third question requested participant comments and/or suggestions, and the top 2 themes in these responses were (1) expressions of gratitude or happiness (81.4%, n = 57) and (2) requests for program extension (7.14%, n = 5).

**Table 4.** Curriculum Evaluation: Qualitative Analysis From Participants (N = 70) of a Community Health Worker-Led Diabetes Program

Evaluation Component	Top 2-3 Themes	Theme Subcategories	Total
What did you like about the diabetes classes?	Educational component	Classes	48.6% (n = 34)
		Nutrition education	
	Team characteristics & factors	Friendliness of staff	40.0% (n = 28)
		Instruction and method of explanation of content	
		Attention provided from staff	
		Treatment of participants with respect and care	
		Motivation & push provided by staff	
		Support provided by staff	
	Everything	N/A	52.9% (n = 37)
What would you want to change about the diabetes classes?	Nothing	N/A	77.1% (n=54)
	Extension of current program components	More time	10.0% (n = 7)
		Program extension	
		More education and information	
	Adjust session schedule	Timing and date options	2.9% (n = 2)
Comments/suggestions	Expressions of gratitude/happiness	N/A	81.4% (n = 57)
	Request for program extension	N/A	7.1% (n = 5)

Participant attendance levels of group visits was high (mean 81.6%, range 79.0%-85.5%).

### CHW Survey

CHWs had high levels of training satisfaction (mean overall score 5.9/6.0). CHWs found that the instructor had expertise and knowledge (5.9/6.0), that objectives were met (5.9/6.0), and that they gained increased knowledge, interest, and usefulness in their work (5.8/6.0). CHWs commented that they will use the course content to improve their health and the health of their families and participants, and they requested content on future topics related to other chronic diseases, such as blood pressure, depression, and cholesterol.

## Discussion

Investigators described TIME’s curricula and provided a descriptive analysis of its acceptability among participants. In addition to improved clinical outcomes previously reported in the TIME studies,^14-17^ participants were very satisfied with their CHW-led group visits and had high attendance levels. CHWs function in a variety of health care roles, interventions, and settings. This study demonstrates a CHW training that is high-quality, step-wise, and evidence-based and the ability for CHWs to implement it in real-world settings. This is essential for improving CHW preparedness, creating sustainable programs, and moving toward national standards in CHW training.^11^

Optimizing CHW training addresses the gaps in CHC care to assist in reducing disparities in Latino(a)s. CHCs represent the safety net of US health care.^34^ CHCs strive to deliver high-quality and culturally competent primary care to populations affected by economic disadvantage, lack of insurance, and health disparities.^34^ Often, CHCs must provide this care in the face of increasing demands and while burdened by limited resources, weak training infrastructures, and logistical challenges.^34,35^ The majority of CHCs report an inadequate supply of clinicians, high turnover rates, and lower levels of clinician and staff satisfaction.^36^ These limitations have a ripple effect that ultimately affects the health of low-income populations that are already at an increased risk for preventable chronic diseases, including diabetes, heart disease, and obesity.^37^ Specifically, Latino(a)s bear a disproportionate burden of diabetes prevalence and incidence in the United States and face a significantly higher risk of disease-related complications.^38-40^ With the reduction of health disparities being a major public health priority, improving diabetes prevention and care for this population represents a critical priority.^41-45^

Recommendations for the development and sustainability of CHWs include clearer definitions of the workforce, training standards, and development of support networks.^9^ Investigators have highlighted that insufficient infrastructure, including national guidelines, limits maximizing full potential effects on participant outcomes. For example, a systematic review evaluating 32 studies on CHW training demonstrated that the scalability of their training is hindered by insufficient evidence on content, teaching/learning strategies, duration, and effectiveness.^46^ However, investigators noted that properly trained CHWs can provide valuable input for cardiovascular disease and type 2 diabetes treatment and prevention.^46^ Another study evaluating CHWs working in disaster resilience showed a similar pattern, where competencies were defined but without the level of specificity needed to develop a comprehensive training curriculum.^47^

A significant strength of this study is the potential to generalize the described curriculum that CHWs taught to participants to other diseases and populations. Specifically, the curriculum provides foundational elements that can guide other investigators in their work, including generalizability to CHWs conducting their role in a variety of medical specialty areas beyond diabetes. For instance, alterations to the 6-month program could generate a curriculum specific to cancer education. The monthly topic areas presented in the curriculum could remain consistent while adjusting the specificity of the content, such as nutrition specific to cancer participants (month 3) or exercise (month 6). Furthermore, the curriculum may also be adapted to other nonmedical personnel, such as lay health advisors, peer health advocates, health auxiliaries, participant navigators, health promoters, and health educators. The modules could also be adapted and translated for use in other languages and cultures. Additional strengths include the introduction of a novel, bilingual curriculum to empower CHWs to educate participants in chronic disease management.

This study is limited by being tested in a specific population (low-income uninsured Latino[a]s in the United States with type 2 diabetes). The findings from the TIME studies may not be applicable to other populations or for participants with different conditions. Some outcomes relied on self-reported data, which can be vulnerable to response bias. Future studies are needed to evaluate acceptance of the curriculum and training in other populations and for other diseases and conditions.

## Conclusions

This study highlights the ability to train CHWs on disease-specific curricula and for them to educate individuals with type 2 diabetes about it. Findings from this study provide a template for future researchers to use these step-wise curricula to provide additional real-world implementation of CHW initiatives with standardized training for other diseases and conditions. This research is a pivotal piece for coming to a national consensus on CHW training and certification, which is needed to maximize CHWs’ roles, improve participant outcomes, and minimize system burdens.
